# Adult white New Zealand rabbit as suitable model for corneal endothelial engineering

**DOI:** 10.1186/s13104-015-0995-1

**Published:** 2015-02-04

**Authors:** Jorge E Valdez-Garcia, Juan F Lozano-Ramirez, Judith Zavala

**Affiliations:** Tecnologico de Monterrey, School of Medicine and Health Sciences, Ophthalmology Research Chair, Monterrey, NL México; Ophthalmology Institute, Tec Salud, Monterrey, NL México

**Keywords:** Cornea, Corneal engineering, Endothelium, Mitosis, New Zealand rabbit

## Abstract

**Background:**

Corneal endothelium engineering is focused in producing transplantable cell sheets to overcome the shortage of corneal graft tissue donors for the treatment of corneal blindness. For this purpose, the use of a proper animal model plays a key role. Corneal parameters of White New Zealand rabbits such as endothelial cell density, central corneal thickness, and corneal diameter decrease with age, similarly as in humans. However, as opposed to humans, they retain the ability to restore their corneal endothelium after injury. Therefore, they are considered as an inappropriate corneal endothelial wound healing model.

**Findings:**

Here we analyze the corneal endothelium mitotic ability of White New Zealand rabbits aged 3, 6, 12 and 18 months, 36 and 72 hours after thermal injury. The highest mitotic activity was observed in the 3-month rabbits 36 h after wounding. Rabbits of 12 months registered decreased mitotic activity and those of 18 months did not show mitotic activity 72 h after injury.

**Conclusions:**

These results propose that rabbits of 18 months represent a suitable model for human corneal endothelium engineering research.

## Findings

### Introduction

The corneal endothelium (CE) is the monolayer of polygonal cells that forms the posterior surface of the cornea facing the aqueous humor [1,2]. Its main function is to maintain an adequate hydration of the cornea through an ATP-bicarbonate pump, providing the clarity the eye needs to execute its visual functions properly. In humans, CE does not have a significant capacity for *in vivo* regeneration, thus making it unable to replace dead or damaged cells. This occurs because human CE cells are arrested in the G1-phase of the cell cycle [3]. When injury occurs, CE cells (CECs) function may be affected leading to corneal opacity and loss of vision that can only be treated by performing a corneal graft. This procedure faces the limited availability of tissue donor and the surgical adverse effects; which include higher incidence of glaucoma, immune graft rejection, and loss of transplanted tissue viability [4-6].

Currently, engineered corneas are being tested in animal models in order to develop new strategies for CE regeneration [7]. The rabbit has been widely used in corneal endothelium studies [8-13] given that it shares characteristics with human CE such as diameter (which allows the use of the same surgical instrumentation as in humans), repair mechanisms, thickness, and composition. Moreover, previous studies report that parameters such as CE density, central corneal thickness, and corneal diameter decrease with age in rabbits, similar to in humans [14,15]. However, the use of rabbits as human CE wound healing model is limited given its ability to restore after injury, making it difficult to establish the effectiveness of the tested treatment. Nevertheless, this is based in studies conducted in rabbits of up to 12 months old in which cell density is measured after CE injury. In this study, we analyzed the number of mitotic figures after injury in rabbits aged from 3 to 18 months in order to address the relationship between the replicative CECs ability and age.

### Material and methods

A total of 16 New Zealand rabbits were used in accordance with the standards set in the Guide for the Care and Use of Laboratory Animals and with the ethical approval of the Internal Committee for the Use and Care of Laboratory Animals from the School of Medicine, Tecnologico de Monterrey. Four age groups were formed: 3, 6, 12 and 18 months. Rabbits were studied with whole cornea histological preparation. All animals were anesthetized with an intramuscular injection of ketamine HCL (30 mg/kg) and xylazine (5 mg/kg), and each animal received two drops of proparacaine prior to wounding. Rabbits were injured in the central cornea for 15 sec with a 2-mm brass cryoprobe cooled to -60°C. Local analgesia with pranoprofen (0.01%) was administered three times per day in each animal after wounding. One-half of the animals were sacrificed at 36 h post-wounding and the other half at 72 h post-wounding. The experimental groups were designated 3/36, 6/36, 12/36, 18/36, 3/72, 6/72, 12/72 and 18/72 (Table 1), with an n = 4 eyes for each experimental group. Given that no standard deviation data for mitoses in CE after thermal wounding from previous published studies were available, this *n* was determined to be an appropriate size by using the resource equation method [16], in which 10 < E > 20.

**Table 1:** **Description of experimental groups**

**Post-wounding sacrifice (h)**	**Age of rabbits (months)**			
	**3**	**6**	**12**	**18**
36	3/36	6/36	12/36	18/36
72	3/72	6/72	12/72	18/72

The time points of sacrifice post-wounding are in the left column and the age of the rabbits in the right columns. The intersection of each time/age represents an experimental group (*n* = 4 eyes).

Both eyes of each rabbit were enucleated and the corneoscleral button was excised. The tissue was fixed for 24 h in acetic acid alcohol. After a second rinse in 50% ethanol, the cornea was stained for 5 min in Harris Hematoxylin solution and rinsed again for 30 sec in 50% ethanol. The cornea was flattened and mounted on a coverslip with the endothelium side up in a water-soluble medium. A #1 cover glass was placed over the cornea. The entire cornea was examined for the presence of mitotic figures. ANOVA was used to analyze difference among the experimental groups and t-test to identify significant differences in the different age groups means.

### Results

**Figure 1:**
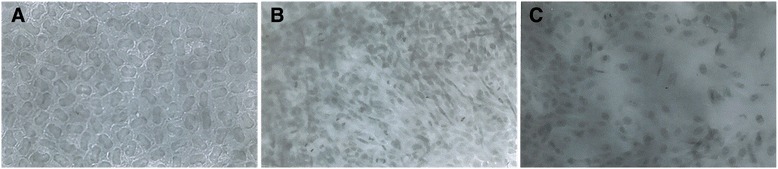
**Flat preparation of the corneal endothelium 36 h after transcorneal freezing. (A)** Transitional zone showing prominent nuclei with a slight increase in distance among them. **(B)** Regenerative zone with mitotic figures and migrating cells. **(C)** Central zone with denuded areas, rare mitotic figures and migrating cells (Hematoxylin, X82.5).

The data for the right and left eyes was pooled for each age group and time point given that there were no statistical differences between them for histological preparations. In all cases, it was possible to identify the wound area with three distinct zones. The outermost area (transitional zone) was characterized by nuclei dispersing and no mitotic figures. In the adjacent zone (regenerative zone) an increase in the number of nuclei and mitotic figures was found. The innermost zone (central), showed scattering of the nuclei, cell migration, and spaces between cells (Figure 1).

**Figure 2:**
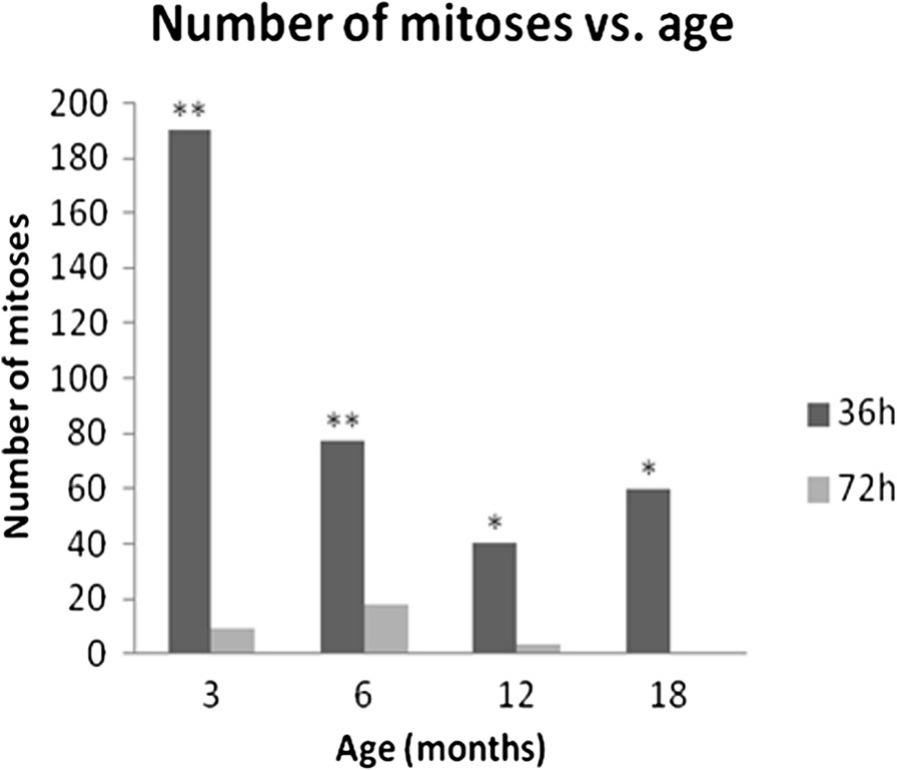
**Number of mitoses found in the different age groups at 36 and 72 h post-injury of the central cornea.** Each data point represents the mean of registered mitoses (*n* = 4). The replicative response was higher for the 3/36 group compared to the older groups (6 - 18/36) at 36 h post-wounding. **p < 0.01, *p < 0.05.

Statistically significant difference among group means was registered as determined by ANOVA test (*p* < 0.05). Mitotic figures found among the different age groups at 36 h decreased considerably from an average of 190 to 40. The highest mitotic activity was found in the 3/36 group with a mean of 190 mitotic figures (Figure 2); this number decreased to 77 in the 6/36 group. Beyond this age, the number of mitoses showed a slight decrease; 40 in the 12/36 group and 60 in the 18/36 group. At 72 h, the average number of mitoses was similar in all the groups.

For all ages, the highest number of mitoses was observed at 36 h, with a significant decrease occurring at 72 h. This was most apparent in the 3/36 vs. 3/72 group (*p* < 0.01). Group 18/72 registered no mitotic figures.

### Discussion

Corneal endothelium bioengineer aims at the production of transplantable cell sheets in order to overcome the shortage of tissue donors in the treatment of corneal blindness. For this purpose, the novel techniques for isolating and harvesting CECs, along with the development of compatible biomaterials to be used as scaffolds, need suitable animal models for testing.

Small mammals such as mouse and rat are often used given that they are easy to handle and offer advantages like high metabolic rates and short life span. However, the surgical techniques required for these animals are different from those used in bigger animals like cats or dogs, and may result in lack of precision and development of modified healing responses. In addition, the mitotic capacity of corneal endothelium *in vivo* appears to be greater in small animals than in humans [17,18]. Cats, dogs, pigs and sheep have been reported to be useful for human corneal research, mainly because of their corneal endothelium limited ability to proliferate *in vivo* [19-22]. Nevertheless, the lack of inbred strains, costs, and handling of bigger animals may difficult the use of these animals in research. Although rabbits have also been widely used for human corneal models, it is thought that the cornea does not fully resemble human corneal endothelium given its regeneration capacity. CE thermal injury is a widely used method to produce CE wound given that it does not damage other corneal structures. In the current study, the mitotic figures of 3-, 6-, 12- and 18-months- old White New Zealand rabbits were analyzed after thermal injury. The results provide evidence of rabbit corneal endothelium regeneration capacity after wound.

The histological analysis revealed different healing zones. The regenerative zone represented the leading edge of the healing process, where the two most important mechanisms of healing occurred: migration and mitosis. Some age differences were found in this zone, the most notable being the presence of extensive mitotic activity at 36 h post-wounding in the 3-month-old rabbits (group 3/36); meanwhile, in older groups (6–18/36), mitotic activity was relatively constant.

Previous reports have shown that rabbit corneal endothelium repairs by cell division and migration. In a study where the regenerative capacity between young cats and rabbits was compared, it was shown that after 24 h post-wounding, the cells of the rabbit corneal endothelium at the margin of the wound were more likely to divide and migrate than those at the peripheral zone [23]. Later, the same group reported that 12-month-old rabbits incorporated tritiated thymidine into the cells at the wound edge to a lesser extent than 6 to 8-week-old rabbits 24 h after transcorneal freezing [24]. This denoted decreased ability of cell division post-wounding in the 12-month-old rabbits. This is in accordance with our results in which 12-month-old rabbits showed decreased mitotic ability after wounding compared with rabbits aged 3 and 6 months. However, 18-month-old rabbits showed no mitotic activity at 72 h post-wounding, making this age group a better model for corneal endothelium engineering research.

In previous studies, it has been shown that parameters such as CECs density, central corneal thickness, and corneal diameter decrease with age in rabbits as in humans. Morita *et al.* [14] found that cell density was lower in rabbits over 12 months. And in 1995, Doughty [15] concluded that a minimum age of 9 months was recommended for the use of rabbits in studies of aged cornea, given the increased polymegathism and decreased cell density. Considering these observations along with our results about the reduced mitotic ability of adult rabbits after wound, as in humans, it may be proposed that rabbits of 18-months-old represent a suitable model for studies of human corneal endothelium engineering.

In summary, our results complement the previous knowledge regarding the similarities the rabbit CE shares with that of human. Our findings showed that the replicative ability of the corneal endothelium found in rabbits demonstrates an inverse relationship with age.
